# Genome Analysis of *BnCNGC* Gene Family and Function Exploration of *BnCNGC57* in *Brassica napus* L.

**DOI:** 10.3390/ijms252111359

**Published:** 2024-10-22

**Authors:** Yue Wang, Qing Chi, Wenjing Jia, Tiantian Zheng, Binghua Li, Lin Li, Ting Li, Rui Gao, Wenzhe Liu, Shenglin Ye, Ruqiang Xu, Hanfeng Zhang

**Affiliations:** 1 National Key Laboratory of Cotton Bio-Breeding and Integrated Utilization, School of Agricultural Sciences, Zhengzhou University, Zhengzhou 450052, China; wangyuer0127@163.com (Y.W.); qchi@zzu.edu.cn (Q.C.); wj210403@gmail.com (W.J.); tiantianzheng1023@163.com (T.Z.); liibinghua@163.com (B.L.); 15993771838@163.com (L.L.); liting082617@163.com (T.L.); gr71025@163.com (R.G.); lwz1102811919@163.com (W.L.); slye1466424924@163.com (S.Y.); xrq65@zzu.edu.cn (R.X.); 2 Henan Key Laboratory of Ion-Beam Green Agriculture Bioengineering, School of Agricultural Sciences, Zhengzhou University, Zhengzhou 450001, China

**Keywords:** genome, CNGC, seed size, *Brassica napus* L.

## Abstract

The cyclic nucleotide-gated ion channel (CNGC), as a non-selective cation channel, plays a pivotal role in plant growth and stress response. A systematic analysis and identification of the *BnCNGC* gene family in *Brassica napus* is crucial for uncovering its biological functions and potential applications in plant science. In this study, we identified 61 *BnCNGC* members in the *B. napus* genome, which are phylogenetically similar to *Arabidopsis* and can be classified into Groups I-IV (with Group IV further subdivided into IV-a and IV-b). Collinearity analysis with other species provided insights into the evolution of *BnCNGC*. By homology modeling, we predicted the three-dimensional structure of BnCNGC proteins and analyzed *cis*-acting elements in their promoters, revealing diverse roles in hormone regulation, growth, and stress response. Notably, overexpression of *BnCNGC57* (*BnaC09g42460D*) significantly increased seed size, possibly through regulating cell proliferation via the MAPK signaling pathway. Our findings contribute to a better understanding of the *BnCNGC* gene family and highlight the potential regulatory role of *BnCNGC57* in the seed development of *B. napus*.

## 1. Introduction

*Brassica napus* (AACC, 2n = 38) is one of the most important oil crops in the world [1]. Its oil supply accounts for about 13% of the world’s vegetable oil and is widely planted in China, Canada, and some European countries [2,3,4]. The cyclic nucleotide-gated channel (CNGC) gene family plays a crucial role in physiological processes such as plant growth and development, defense response and signal transduction [5,6,7]. CNGCs were initially identified during the screening process for the calmodulin-binding transporter (HvCBT1) in barley [8]. Subsequently, CNGCs were also detected in various other plant species, including *Arabidopsis*, rice, tomato, and *B*. *oleracea* [9,10,11,12]. Currently, these channels are classified as a large protein family. Research on the CNGC family has found that plant CNGC genes may be functionally differentiated in a group-dependent manner. For example, the *Arabidopsis* CNGC family is divided into four major groups (I–IV) based on phylogenetic relationships, with Group IV further divided into subgroups IV-a and IV-b [9]. Plant CNGCs proteins have complex conserved domains and subunit structures, with a core structure consisting of six transmembrane domains (S1–S6) and a pore-like region (P loop) between S5 and S6, which may affect ion selectivity. The cytoplasmic C-terminus contains a cyclic nucleotide-binding domain (CNBD) [13]. CNBD contains two conserved domains; namely, the phosphate-binding Cassette (PBC) and the calmodulin-binding domain (CaMBD) ‘hinge’ region, which is highly conserved in the structure [13]. CNGCs are cation channels with varying degrees of ion conduction selectivity. The activity of CNGCs is regulated by reversible binding of cyclic nucleotides (cNMP: adenosine 3′,5′-cyclic monophosphate (cAMP) and guanosine 3′,5′-cyclic monophosphate (cGMP)) or calmodulin (CaM) to CNBD, leading to conformational channel opening [6,14,15]. The increase in intracellular Ca^2+^ levels activates calcium receptors such as CaM and calcium-dependent protein kinases (CDPKs), which bind to CaMBD and inhibit the binding of cNMP to CNBD, thereby suppressing the activity of CNGCs [16,17,18]. In addition, CNGCs are located on various organelle membranes such as plasma membrane, chloroplast membrane, vacuolar membrane, or nuclear membrane [12,19,20,21]. The CNGC gene family has been found in multiple plant species. For example, 20 CNGC genes were identified in *Arabidopsis*, 26 in *B*. *rapa*, 29 in *B*. *oleracea*, and 16 in rice [13,22,23].

Previous studies have shown that CNGCs play an important role in plant growth and development, participating in various processes such as polar apical growth of pollen, apical growth of root hairs, and leaf senescence [24,25,26]. The tissue-specific expression pattern analysis of tobacco CNGCs showed that 23 *NtCNGC* genes were differentially expressed in different tissues [27]. Research has shown that *AtCNGC7/8/16/18*, members of the *Arabidopsis* CNGC family, are specifically expressed in pollen and participate in the polar growth of pollen tubes [28,29,30]. The *AtCNGC7/CNGC8* double mutation can lead to male infertility, and it plays a certain role in pollen germination and male fertility [31]. *AtCNGC16* is beneficial for the development of *Arabidopsis* pollen grains under hot environmental conditions [32]. *AtCNGC18* has Ca^2+^ channel activity. When Ca^2+^ activates *AtCPK32*, *AtCPK32* interacts with CNGC18 and activates it through phosphorylation. This activated state promotes Ca^2+^ entry into the top of the pollen tube, ultimately promoting polar growth of the pollen tube [23]. There is an interaction between the intracellular C-terminus of *AtCNGC14* and *AtCaM7*, which inhibits the channel activity of *AtCNGC14* by binding to *AtCaM7*. The two regulate the normal polar growth of root hairs by precisely controlling the Ca^2+^ level at the tip of the root hairs [33]. The application of NO donors to the *AtCNGC2*-deficient mutant *dnd1* can effectively rescue many early aging phenotypes of *dnd1* plants, demonstrating the involvement of *AtCNGC2* in signal transduction related to the plant aging signaling cascade [24]. Two subgroups, Group IV components a and b, are involved in plant thermotolerance among the Group IV-b members in *Arabidopsis* and rice [34,35]. The majority of *BnCNGCs* expressed in Group Ⅳ-b in *B. napus* is strongly induced by SA treatment and *B. sclerotinorum* infection and plays an important role in SA-mediated sclerotiorum response in *B. rapa* [36]. In addition, in the model plant *Arabidopsis*, *AtCNGC2* and *AtCNGC4* together form Group IV-b and play an important role in calcium signal transduction related to various physiological processes such as pathogen defense, growth and development, and heat tolerance [37,38,39]. The role of CNGCs in plant defense was first proposed in the study of *AtCNGC2 Arabidopsis*-deficient mutants [40]. CNGC2 not only participates in plant immunity but also participates in physiological processes such as plant flowering response and leaf senescence [37,41]. However, research on CNGCs involved in regulating plant growth and development is mainly concentrated on model plants, and the functional research of CNGCs in other plant growth and development has not been reported.

*Brassica napus* (AACC, 2n = 38) is an allopolyploid crop formed by natural hybridization and chromosome doubling of *Brassica rapa* (AA, 2n = 20) and *Brassica oleracea* (CC, 2n = 18) [42]. In this study, we conducted a whole-genome identification of the CNGC family genes in *B. napus* and performed a detailed analysis of their collinearity relationships, three-dimensional structures, protein transmembrane structures, and cis-acting elements. In addition, to further investigate the role of *BnCNGC* genes in the development of *B*. *napus*, we constructed overexpression transgenic lines of the *BnCNGC57* (*BnaC09g42460D*) gene, which has a high degree of homology with *AtCNGC2*. Notably, these transgenic lines overexpressing *BnCNGC57* (*BnaC09g42460D*) showed significant positive effects on seed size and yield. Furthermore, we analyzed the potential regulatory mechanism of the *BnCNGC57* (*BnaC09g42460D*) gene in seed development through transcriptomics. This study lays a foundation for in-depth research on the regulatory role of CNGCs in plant growth and development responses.

## 2. Results

### 2.1. Identification and Characterization of CNGCs Family Genes in B. napus

The BLASTP alignment, along with the analysis of protein domains and CNGC-specific motifs, revealed the presence of 61 *BnCNGC* genes within the *B. napus* genome. Phylogenetic tree analysis corroborated previous studies in *Arabidopsis*, *B. oleracea*, *B*. *rapa*, and other related species, classifying these *CNGCs* into four distinct groups (I, II, III, and IV). Group IV was further subdivided into two subgroups (IV-a and IV-b). Based on the phylogenetic tree outcomes, the 61 CNGC members were designated as *BnCNGC1* through *BnCNGC61* (Table 1).

**Figure 1:**
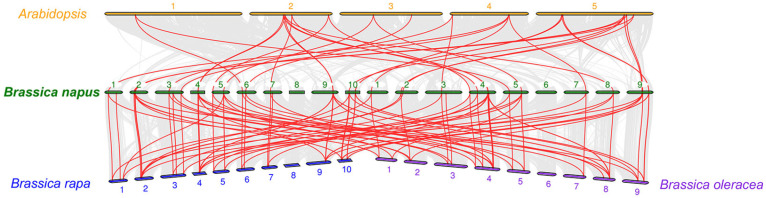
Synteny analysis of the *CNGC* genes. Gray lines in the background indicated the collinear blocks within *B. napus* and other plant genomes, and the red lines highlighted the syntenic *BnCNGC* gene pairs.

To further infer the evolutionary pathway of the *BnCNGC* family, collinearity analysis was conducted using *Arabidopsis*, *B. oleracea*, and *B. rapa* and the CNGC family members from *B. napus*. As shown in Figure 1, there are 34 collinearities between *B. napus* and *Arabidopsis*. This indicates that during the evolutionary process, there is a certain degree of genetic association between *B. napus* and *Arabidopsis* at some gene levels. Possibly, due to a common ancestor, some genes have been retained and play similar functions in different species during the long evolutionary journey.

However, the collinearity between *B. napus* and *B. oleracea* and *B. rapa* is significantly more than that with *Arabidopsis*, further indicating a closer genetic relationship. This is because *B. napus* is an allopolyploid crop formed by natural hybridization and chromosome doubling of *B. rapa* and *B. oleracea*. During the evolutionary process, the genes of *B. rapa* and *B. oleracea* are combined into *B. napus*, making their gene similarity higher. The existence of this collinearity also implies that CNGC family genes may have experienced events such as gene duplication and recombination during evolution, thus forming similar gene structures and functions in different species. It indicates that CNGC family genes are highly conserved during evolution. This high degree of conservation may be due to the crucial role of CNGC genes in physiological processes such as plant growth and development, defense responses, and signal transduction. Natural selection tends to retain the functions of these key genes to ensure the survival and reproduction of plants.

### 2.2. Homology Modeling of BnCNGC Genes in Group IV-b

**Figure 2:**
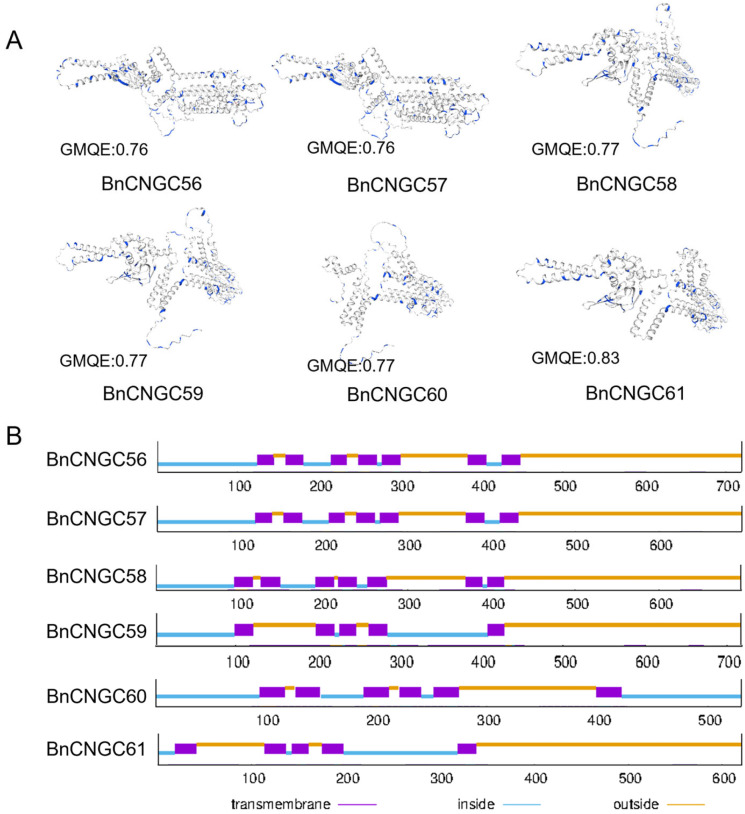
The 3D structure modeling and protein transmembrane structure prediction of BnCNGC proteins in Group IV-b. (**A**) The 3D structure modeling of BnCNGC proteins; blue represents Ser/Thr site. (**B**) Prediction of transmembrane structure of BnCNGC proteins. Purple represents transmembrane, blue line represents inside, and yellow represents outside.

We conducted 3D structural homology modeling and protein transmembrane structure prediction for the Group IV-b CNGC genes of *B. napus* that are homologous to *Arabidopsis*. The 3D structure of the Group IV-b BnCNGC proteins was predicted using homology modeling techniques. The Global Model Quality Estimate (GMQE) values were all greater than 0.75, suggesting that the predictions are reliable. The findings indicate that *BnCNGC56*, *BnCNGC57*, and *BnCNGC61* share similar three-dimensional structures, and *BnCNGC58* and *BnCNGC59* have identical 3D structural models, with *BnCNGC58* and *BnCNGC59* being homologous to *AtCNGC2* (Figure 2A). To predict the transmembrane structures of the Group IV-b BnCNGC proteins, *BnCNGC56*, *BnCNGC57*, and *BnCNGC58* were found to have seven transmembrane structures, while *BnCNGC59* and *BnCNGC61* have five, and *BnCNGC60* has six (Figure 2B). A statistical analysis of the Ser/Thr sites outside the transmembrane domains revealed that *BnCNGC58* possesses the highest number of Ser/Thr sites outside the transmembrane domain, with 55 sites, whereas *BnCNGC60* has the fewest, with 18 sites (Table S1). Additionally, *BnCNGC57*, *BnCNGC56*, and *BnCNGC59* have 45, 44, and 50 Ser/Thr sites, respectively, outside their transmembrane domains (Table S1). The abundance of Ser/Thr sites outside the transmembrane domains suggests that BnCNGC proteins can undergo phosphorylation at regulatory sites, serving as binding and regulatory targets for kinases. This enhances their complexity and flexibility during signal transduction and plays a more critical role in signaling and regulation.

### 2.3. Analysis of cis-Regulatory Elements

**Figure 3:**
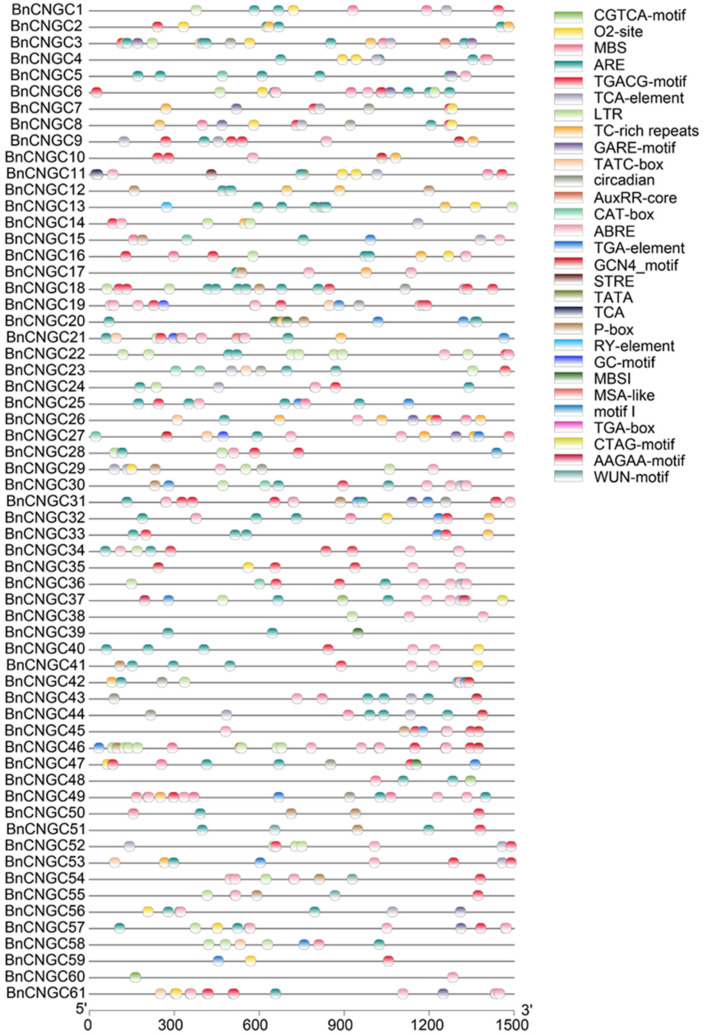
Predicted *cis*-acting regulatory elements in *BnCNGC* promoters. Promoter regions 1500 bp upstream of the *BnCNGC* genes translation start sites were analyzed by PlantCARE.

The *cis*-acting elements in the 1500 bp promoter region upstream of the translation start site of the *B. napus* CNGC genes were predicted by using PlantCARE software (http://bioinformatics.psb.ugent.be/webtools/plantcare/html/, accessed on 30 January 2024). Multiple cis-regulatory elements related to salicylic acid-responsiveness element (TCA-element), abscisic acid-responsiveness element (ABRE), MeJA-responsiveness element (CGTCA-motif), auxin-responsive element (TGA-element), gibberellin-responsive element (GARE-motif), MYB binding site involved in drought-inducibility (MBS), and defense and stress responsiveness elements (TC-rich repeats) were found (Figure 3). The TCA element indicates that the *BnCNGC* genes may be involved in plant resistance to pathogens. The ABRE element indicates that it may be regulated by the ABA signaling pathway under adverse conditions such as drought. The CGTCA-motif element indicates that the *BnCNGC* genes may be involved in plant defense and development. The TGA element indicates that the *BnCNGC* genes may play a role in auxin-regulated growth and development. The GARE-motif shows that it may synergistically participate in growth regulation with gibberellin signals. The MBS and TC-rich repeats elements further confirm the potential role of the *BnCNGC* genes in stress and defense responses.

In addition, some elements are also involved in the regulation of cell cycle regulation elements (MCA-like) and the seed-specific regulation element (RY-element) (Figure 3). The MCA-like suggests that the *BnCNGC* genes may play a role in the regulation of the cell cycle and affect plant cell division and growth. The RY-element indicates that the *BnCNGC* genes may have specific functions in seed development and expression.

Besides the basic cis-acting elements TATA-box and CAAT-box, most other elements are related to plant hormone signal transduction and stress response. This proves that the *BnCNGC* family may be involved in various stress responses and hormone regulation of plants (Figure 3, Table S2). The TATA-box and CAAT-box are essential for initiating gene transcription. The presence of other elements related to hormone signal transduction and stress response further emphasizes the importance of the *BnCNGC* gene family in the process of plants responding to different environmental stimuli and growth and development. These genes may regulate plant physiological processes by responding to different hormone signals and stressors to adapt to complex and changeable environmental conditions.

### 2.4. Overexpression of BnCNGC57 Significantly Increased the Seed Size

**Figure 4:**
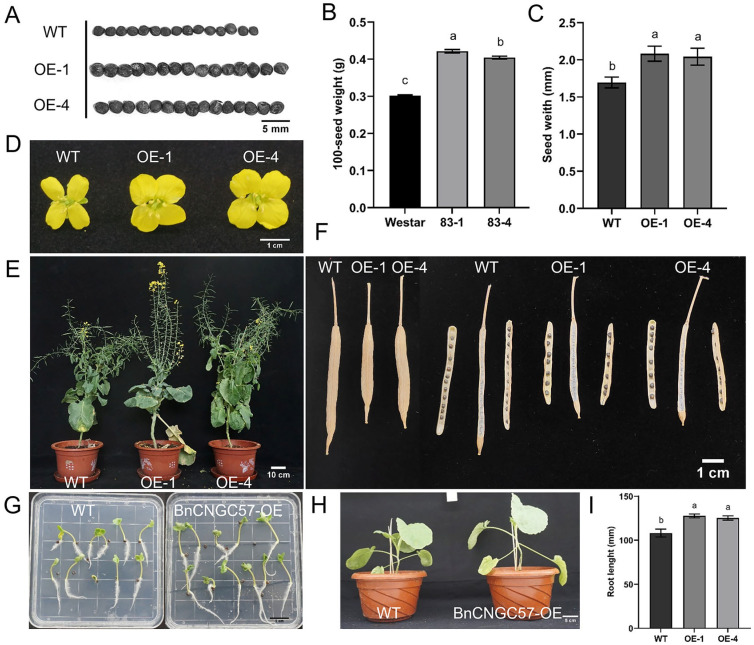
Agronomic traits of *BnCNGC57* gene overexpression lines. (**A**) Seed size (scale, 5 mm). (**B**) 100-seed weight. (**C**) Seed width. (**D**) Flower phenotype (scale, 1 cm). (**E**) Phenotype of *BnCNGC57* overexpressing rapeseed plants (scale, 10 cm). (**F**) Silique phenotype (scale, 1 cm). (**G**) Phenotype of seeds germinated for 3 days (scale, 1 cm). (**H**) Phenotype of plant seedlings (scale, 5 cm). (**I**) Root length of seedlings germinated for 10 days. “WT” is wild-type plant Westar, and “OE-1” and “OE-4” are *BnCNGC57*-OE lines. Values and error bars represent mean ± SD (n = 3), and letters indicate significant differences by one-way ANOVA statistical analysis (*p* < 0.05).

To evaluate the phenotype of transgenic lines, we selected lines with relatively high expression levels (Supplementary Figure S3). Relative to the wild type, both overexpression lines displayed a substantial enhancement in seed size (Figure 4A), with a notable increase in 100-grain weight of 39.62% and 33.89%, respectively (Figure 4B), and an in-seed width of 22.89% and 20.53%, respectively (Figure 4C). Moreover, we examined additional agronomic traits closely associated with seed development in the two *BnCNGC57* overexpressing transgenic lines. Seed germination in the transgenic lines paralleled that of the wild type (Figure 4G), yet the seedlings exhibited accelerated growth, characterized by longer roots and a more robust plant stature (Figure 4G–I). The *BnCNGC57* overexpressing transgenic lines exhibited earlier flowering than wild-type plants and a significant enlargement in flower size (Figure 4D). Furthermore, the horn width was notably broader compared to the wild type, and the number of grains per horn was also significantly reduced (Figure 4E,F). These findings collectively suggest that the overexpression of the *BnCNGC57* gene results in larger seeds and increased grain weight.

### 2.5. Transcriptome Analysis

**Figure 5:**
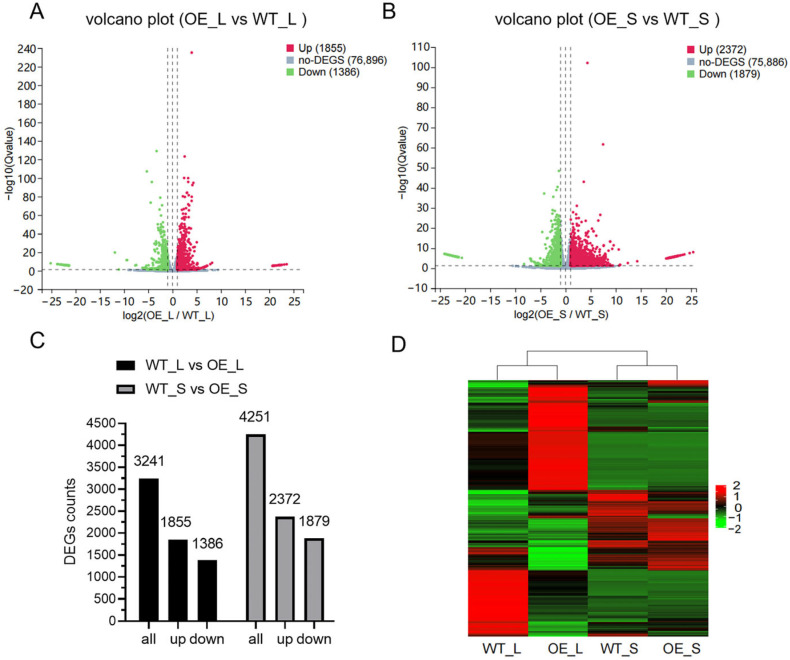
Overview of RNA-seq data of transgenic lines overexpressing *BnCNGC57* relative to the wild type (WT). (**A**) Volcano plot of differentially expressed genes (DEGs) in leaves. (**B**) Volcano plot of DEGs in seeds. There are three vertical dashed lines on one side of the y-axis. The leftmost vertical dashed line represents Log2FC = −1. Genes on the left side of the dashed line with down-regulated expression (Log2FC ≤ −1) are marked as blue dots. The middle vertical dashed line corresponds to a log₂ fold change of 0. The rightmost vertical dashed line represents Log2FC = 1. Genes on the right side of the dashed line with up-regulated expression (Log2FC ≥ 1) are marked as red dots. The horizontal axis represents the fold change of differential expression, and the vertical axis represents the significance level of gene expression differences. (**C**) Number of DEGs. (**D**) Heatmap of DEGs.

In this study, leaves of the transgenic line overexpressing *BnCNGC57* (*BnaC09g42460D*) (*BnCNGC57*-OE) and wild-type plant Westar, as well as seeds at 20 days after fertilization (20DAP) were sampled separately. This study involved a total of 12 samples, including three biological replicate samples for each treatment. Through transcriptomic analysis, we aim to understand the impact of overexpression of the *BnCNGC57* gene on the transcription level of leaves and seeds of *B. napus*. High-throughput sequencing was performed on the BGISEQ-500 platform, yielding an average of 4.42 × 10^7^ clean reads per sample. After removal of adaptor sequences and low-quality reads, the total length of clean reads reached 8.07 × 10^10^ nt; the Q30 base ratio was higher than 92.92%. More than 79.48% of the clean data were aligned to the *B. napus* reference genome (Table S3). The screening condition for differentially expressed genes (DEGs) were set to |log2 (Fold Change)| ≥ 1, and FDR ≤ 0.05. The analysis of seeds overexpressing *BnCNGC57* revealed substantial differences in gene expression patterns compared to wild-type seeds (Supplementary Figure S4). In the volcano plot, genes with upregulated expression (Log2FC ≥ 1) are denoted by red dots, while those with downregulated expression (Log2FC ≤ −1) and no significant differential expression (|Log2FC| < 1) are indicated by blue and black dots, respectively (Figure 5A,B). The horizontal axis signifies the fold change of differential expression, and the vertical axis represents the significance level of gene expression differences.

Transcriptome analysis yielded 3241 differentially expressed genes between overexpressing *BnCNGC57* transgenic rape and wild-type leaf tissue, with 1386 downregulated and 1855 upregulated genes. Furthermore, 4251 differentially expressed genes were identified between overexpressed *BnCNGC57* transgenic rape and wild-type seeds, comprising 1879 downregulated and 2372 upregulated genes (Figure 5C). To provide a more intuitive representation of the expression patterns of these differentially expressed genes (DEGs), we employed the heatmap tool to generate a cluster heat map (Figure 5D). The horizontal axis of the heat map corresponds to various differentially expressed genes, and the vertical axis represents individual samples. Color coding is utilized to reflect gene expression levels, with red indicating high expression and green indicating low expression. Clustering analysis revealed that overexpressing *BnCNGC57* transgenic lines and wild-type (WT) samples exhibited a significant divergence in gene expression profiles. Expression box line diagram also shows that there are significant differences in the gene expression levels in the leaf and seed tissues of the overexpression lines compared with WT (Supplementary Figure S5). Notably, the majority of genes in the leaves of overexpressing *BnCNGC57* transgenic lines displayed elevated expression levels.

**Figure 6:**
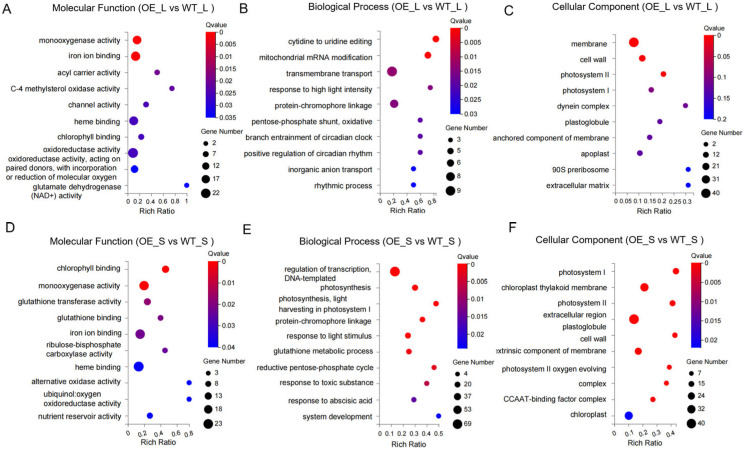
GO enrichment analysis of DEGs in transgenic lines overexpressing *BnCNGC57* relative to the wild type (WT). (**A**) GO enrichment analysis of molecular function of DEGs in leaves. (**B**) GO enrichment analysis of biological process of DEGs in leaves. (**C**) GO enrichment analysis of cellular component of DEGs in leaves. (**D**) GO enrichment analysis of molecular function of DEGs in seeds. (**E**) GO enrichment analysis of biological process of DEGs in seeds. (**F**) GO enrichment analysis of cellular component of DEGs in seeds.

In order to better understand DEGs, Gene Ontology (GO) enrichment analysis was conducted from molecular function, biological process, and cell component classification systems, including the top 10 biological processes in the GO enrichment results of differentially expressed genes in leaves and seeds. Compared to the wild type, leaves of *BnCNGC57* transgenic lines exhibited a significant enrichment in monooxygenase activity (GO: 0004497), iron–ion binding (GO: 0005506), heme binding (GO: 0020037), and oxidoreductase activity (GO: 0016491) (Figure 6A). In terms of biological processes, they were enriched in transmembrane transport (GO: 0055085), protein–chromophore linkage (GO: 0018298), and plant-type cell wall organization (GO: 0009664), among others (Figure 6B). At the cellular component level, they showed enrichment in membrane (GO: 0016020), cell wall (GO: 0005618), photosystem II (GO: 0009523), and photosystem I (GO: 0009522) (Figure 6C). Similarly, seeds from *BnCNGC57* overexpressing transgenic lines were predominantly enriched in chlorophyll binding (GO: 0016168), monooxygenase activity (GO: 0004497), heme binding (GO: 0020037), and oxidoreductase activity (GO: 0016491) (Figure 6D). In biological processes, they were enriched in regulation of transcription, DNA-templated (GO: 0006355), photosynthesis (GO: 0015979), protein–chromophore linkage (GO: 0018298), glutathione metabolic process (GO: 0006749), and additional processes (Figure 6E). At the cellular component level, they were enriched in photosystem II (GO: 0009523), photosystem I (GO: 0009522), cell wall (GO: 0005618), chloroplast thylakoid membrane (GO: 0009535), and extracellular region (GO: 0005576) (Figure 6F). These processes are frequently associated with redox reactions and various biological processes, including transport, photosynthesis, transcription regulation, and changes in cellular components. Such alterations contribute to the promotion of plant growth, development, and seed morphological formation.

**Figure 7:**
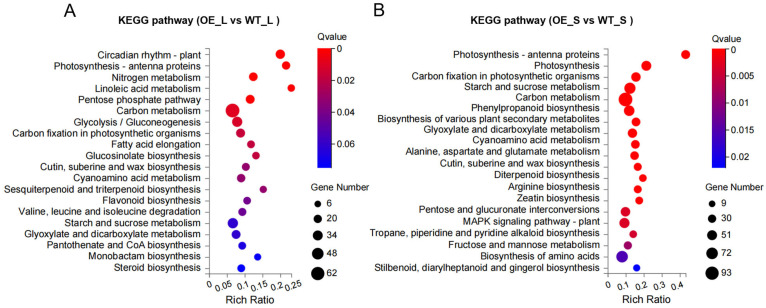
KEGG enrichment analysis of DEGs in transgenic lines overexpressing *BnCNGC57* relative to the wild type (WT). (**A**) KEGG enrichment analysis of DEGs in leaves. (**B**) KEGG enrichment analysis of DEGs in seeds.

Furthermore, the metabolic pathways associated with differentially expressed genes (DEGs) from the leaves and seeds of *BnCNGC57* overexpressing transgenic lines were analyzed using KEGG enrichment analysis. The findings indicate that the metabolic pathways of DEGs in leaves are predominantly enriched in energy metabolism (including photosynthesis-antenna proteins, nitrogen metabolism, carbon fixation in photosynthetic organisms), carbohydrate metabolism (such as the pentose phosphate pathway, Glycolysis/gluconeogenesis, starch and sucrose metabolism), and lipid metabolism (including linoleic acid metabolism, fatty acid elongation, cutin, suberine, and wax biosynthesis) (Figure 7A). In seeds, the metabolic pathways of DEGs are mainly enriched in energy metabolism (such as photosynthesis-antenna proteins, carbon fixation in photosynthetic organisms, photosynthesis), carbohydrate metabolism (including carbon metabolism, starch and sucrose metabolism, glyoxylate and dicarboxylate metabolism), and amino acid metabolism (such as alanine, aspartate and glutamate metabolism, arginine biosynthesis) (Figure 7B). Additionally, the MAPK signaling pathway was also significantly enriched in plant metabolic pathways. From these results, it is evident that the overexpression of *BnCNGC57* markedly enhances the efficiency of energy metabolism, carbohydrate metabolism, and lipid metabolism in transgenic rapeseed plants, and facilitates the synthesis and utilization of amino acids. Collectively, these metabolic alterations impact the growth and development of the transgenic plants, particularly during the stages of seed formation and accumulation. These changes may represent crucial factors that contribute to seed enlargement.

## 3. Discussion

### 3.1. Identification of the CNGC Family Genes in B. napus

CNGC, as a non-selective cation channel, is implicated in Ca^2+^ signal transduction, playing a crucial role in modulating a variety of plant signaling pathways, growth and development processes, as well as responses to various environmental stresses [5,10,43]. Currently, researchers have pinpointed numerous members of the CNGCs family in various plants, including *Arabidopsis*, wheat, rice, maize, and pear [5,44,45,46]. Previous preliminary identification and expression analysis of CNGCs family members in *B. napus* have been conducted, yet functional studies on CNGCs in rapeseed remain underdeveloped [36]. In this study, we identified the CNGC gene family in *B. napus* and identified 61 CNGC genes in agreement with previous reports [36]. Genetic evolution analysis reveals that these genes bear similarities to those of *Arabidopsis* and its progenitors, and are categorized into four main groups (I, II, III, IV) along with two subgroups (IV-a and IV-b) [36,47,48]. It is found that the members of the Group IV are mainly involved in plant immunity and cell death, and they play important roles in various physiological processes such as plant growth and development and pathogen defense [49,50,51,52]. In previous studies, members of Group IV-b, CNGC2, and CNGC4 can form heteromeric channels to regulate Ca^2^⁺ influx and play important roles in plant pollen development and pathogen defense.

In this study, we elucidated the 3D structure of the CNGC protein within the Group IV-b category. A substantial number of Ser/Thr residues located outside the protein’s transmembrane domain facilitate the BnCNGC protein’s enhancement of its role in signal transduction by enabling phosphorylation of regulatory sites (Figure 2A,B). For instance, the calcium ion channel CNGC20 modulates channel activity and protein stability via phosphorylation of the receptor-like kinase PSY1R and the cytosolic receptor kinase CRPK1 [53]. *Cis*-acting elements within gene promoter regions play a crucial role in the regulation of gene expression [54]. Upon analyzing the promoter cis-acting elements, it was discovered that the promoter region of *BnCNGCs* is rich in response elements for various phytohormones, including ABA, IAA, JA, GA, SA, and others. Additionally, it contains elements responsive to stress conditions such as drought and low temperatures (Figure 3). This suggests that the *BnCNGC* genes may play a role in the signaling and regulation of phytohormone pathways and stress responses. Notably, genes such as *CNGC2*, *CNGC4*, *CNGC9*, *CNGC14*, *CNGC16*, and *CNGC20* have been confirmed to be involved in the regulation of cold stress in plants [35,53,55,56,57].

### 3.2. The Overexpression of BnCNGC57 Markedly Enhanced Seed Size

In *Arabidopsis*, *AtCNGC2* and *AtCNGC4* together form Group IV-b. The role of CNGCs in plant defense was first proposed in the study of defective mutants of *AtCNGC2* [40]. CNGC2 was initially recognized as a crucial element of the hypersensitive response (HR), a swift form of localized cell death triggered by an avirulent pathogen [40]. HR serves as a crucial self-defense mechanism in plants, enabling them to safeguard entire leaves through localized cell death at the point of infection [58,59]. The *AtCNGC2* mutant, designated *dnd1* (defense, no death), exhibited a phenotype characterized by stunted plant growth, diminished fertility, and postponed flowering [56,60,61]. However, this particular gene has received limited attention in *B. napus* thus far. Consequently, in this study, we have generated transgenic lines overexpressing *BnCNGC57*, which exhibit a high degree of homology to *AtCNGC2*. Remarkably, the *B. napus* transgenic lines overexpressing *BnCNGC57* display a distinct phenotypic contrast compared to the phenotype observed in *Arabidopsis* mutant plants. Specifically, these transgenic *B. napus* lines exhibit phenotypically accelerated growth rates, more robust root development, enhanced plant architecture, and an earlier onset of flowering (as depicted in Figure 4E,G,H). These observations align seamlessly with previous phenotypic reports documenting the effects of this gene in *Arabidopsis* [40,56]. Furthermore, this investigation revealed that seeds from *B. napus* plants overexpressing *BnCNGC57* exhibited larger sizes and a significant increase compared to those of wild-type plants (Figure 4A–C). This suggests that *BnCNGC57* positively influences the regulation of rapeseed seed size, a phenomenon not previously documented in the scientific literature. Our observations indicated that while the overexpressing *BnCNGC57* lines had larger seeds, they experienced an earlier flowering period, resulting in shorter plants and a subsequent reduction in the number of seeds per harvest (Figure 4D,F). These results imply that *BnCNGC57* might directly or indirectly influence the development of horn-like structures and other plant organs.

### 3.3. Transcriptome Analysis Reveals the Mechanism of BnCNGC57 Gene in Regulating Seed Size of Rapeseed

In *Arabidopsis*, the *AtCNGC2* gene has been confirmed to play a role in regulating plant growth and flowering characteristics; however, its involvement in seed development has not yet been reported [26,40]. In this study, seeds from transgenic lines overexpressing the *AtCNGC2* homolog *BnCNGC57* exhibited a significant increase in size. At the transcriptomic level, the biological functions of the overexpressing *BnCNGC57* transgenic lines were elucidated in various tissues through Gene Ontology (GO) analysis. In leaves, GO terms were notably enriched in biological processes associated with photosynthesis, cell wall structure, transmembrane transport, energy metabolism, and biosynthesis (Figure 6A–C). Consequently, this study hypothesized that the overexpression of *BnCNGC57* could indirectly stimulate the growth and development of rapeseed seeds by enhancing redox and binding functions, optimizing cell wall and membrane structures, and improving the overall physiological state and metabolic network in leaves. In seeds, GO terms were primarily enriched in processes related to photosynthesis, cell wall and chloroplast thylakoid membrane structure, and transcriptional regulation (Figure 6D–F). This enrichment may facilitate the growth and development of photosynthesis, enhance the morphological formation and material accumulation of seeds, and improve the antioxidant capacity while optimizing the cellular component structure. In this study, KEGG pathway analysis revealed that energy metabolism, carbohydrate metabolism, and amino acid metabolism-related pathways were highly enriched in overexpressing *BnCNGC57* transgenic plants (Figure 7A,B). DEGs in both the leaves and seeds of overexpressing *BnCNGC57* were significantly enriched in the energy metabolism and carbohydrate metabolism pathways. The proper functioning of energy metabolism is essential for supporting plant growth and development. Carbohydrates serve as crucial energy sources that enable plants to perform a variety of life processes [62,63]. Enhanced energy metabolism, active carbohydrate accumulation, and seed morphology are intimately linked to material accumulation [64].

Furthermore, the MAPK signaling pathway, also known as the plan pathway, was found to be significantly enriched in the overexpressed *BnCNGC57*. The mitogen-activated protein kinase (MAPK) pathway is a crucial component of cellular signaling, serving as a process for signal cascade amplification. It involves a series of three kinases that are sequentially activated through phosphorylation: MAPK kinase kinase (MAPKKK), MAPK kinase (MAPKK), and MAPK. This cascade amplifies and transmits signals downstream, thereby regulating growth and developmental processes [65]. Previous studies have reported that *DSG1* mutant in rice, encoding *OsMAPK6*, located downstream of *OsMKK4* and *OsMKKK10*, phosphorylated downstream proteins to transmit and amplify signals to regulate cell proliferation in the adult shell, thus affecting cell size and then regulating grain size [66,67,68]. The overexpression of *BnCNGC57* in transgenic plants leads to an increased seed size, potentially through several mechanisms. The *BnCNGC57* gene not only enhances the light capture efficiency and photosynthetic product synthesis, thereby increasing the energy metabolism level, but also promotes the pentose phosphate pathway, glycolysis/gluconeogenesis, and starch and sucrose metabolism, among other carbohydrate metabolism pathways. Furthermore, it facilitates the formation of seed morphology and the accumulation of materials. Additionally, the gene triggers the activation of the MAPK signaling pathway, which in turn regulates cell proliferation and ultimately modulates grain size.

## 4. Materials and Methods

### 4.1. Identification and Collinearity Analysis of CNGC in B. napus

The genome sequences of *B. napus* were retrieved from the Brassicaceae (BRAD, http://brassicadb.cn/, accessed on 16 January 2024) database [69]. The known CNGC sequences of *Arabidopsis* were downloaded from the TAIR (https://www.arabidopsis.org/, accessed on 16 January 2024) databases and used as query sequences in the Basic Local Alignment Search Tool (BLAST) program to search for CNGC genes in *B. napus* genome. Members of the CNGC gene family with characteristic domains (ITP: PF00520\PF07885 and CNBD: PF00027) were identified from the PFAM database [70] (https://pfam.xfam.org/, accessed on 19 January 2024). To reconfirm sequences where PFAM failed to detect or only partially identified the characteristic domains, the multi-domain database analysis platform INTERPRO [71] (https://www.ebi.ac.uk/interpro/, accessed on 20 January 2024) was utilized, resulting in the validation of 79 eligible CNGC protein sequences in *B. napus*. Subsequently, the 79 BnCNGC protein sequences alongside 20 AtCNGC protein sequences were established by constructing a phylogenetic tree via the neighbor-joining method using MEGA 6.06 software [72]. This analysis further refined the initial result, ultimately leading to the identification of 61 definitive members of the CNGC family in *B. napus.* CNGC gene collinearity analyses were analyzed using the MCScanX tool (Multiple Collinearity Scan) (https://www.rcac.purdue.edu/software/mcscanx, accessed on 25 January 2024) with a set of parameters and visualized by the Du-al Systeny plotter in TBtools-Ⅱ (v2.119) [73].

### 4.2. Homology Modeling of BnCNGCs 3D Structure

The 3D structure of the protein is important for understanding its function. We conducted 3D structural homology modeling and protein transmembrane structure prediction for the Group IV-b CNGC genes of *B. napus* that are homologous to *Arabidopsis*. The Swiss-Model interactive tool (https://swissmodel.expasy.org/interactive/, accessed on 30 January 2024) was used to predict the 3D structure of the BnCNGC proteins [74,75]. Additionally, the transmembrane of BnCNGC proteins was predicted using the online tool TMHMM-2.0 [76] (https://services.healthtech.dtu.dk/services/TMHMM-2.0/, accessed on 30 January 2024).

### 4.3. cis-Acting Elements Analysis

The *cis*-acting elements in the promoters of the *BnCNGC* gene family were analyzed using the online software PlantCARE (http://bioinformatics.psb.ugent.be/webtools/plantcare/html/, accessed on 30 January 2024) [77].

### 4.4. Agrobacterium-Mediated Transformation of Rapeseed Hypocotyl

The *B. napus* cultivar Westar is used for Agrobacterium-mediated hypocotyl genetic transformation of rapeseed to generate *BnCNGC57* overexpression (*BnCNGC57*-OE) plants. Through gateway technology, the cDNA of *BnCNGC57* is first ligated to the entry vector pGWC, and then introduced into the binary expression vector pMDC83 through an LR reaction to obtain the constitutive pMDC83-*BnCNGC57* overexpression vector. Then, it is transformed into the Agrobacterium strain *GV3101* according to the method in [78]. Using the hypocotyl of *B. napus* cultivar Westar as the explant, the pMDC83-*BnCNGC57* overexpression vector is transformed into the hypocotyl of *B. napus* cultivar Westar by the Agrobacterium infection method. The infected hypocotyls are screened on MS medium supplemented with 4 mg/L hygromycin and 300 mg/L timentin. During the transformation process, based on the method used in our laboratory in the early stage, 15 mg/L thymol is added to reduce explant browning and increase the differentiation rate [79]. The regenerated seedlings are domesticated in hydroponic solution for 1 week and then transplanted into pots (Supplementary Figure S1). Genomic DNA is extracted from one-month-old leaves, and gene-specific primers are used to amplify fragments containing the *BnCNGC57* gene and the *Hyg* gene sequence to verify the presence of transgenes (Supplementary Figure S2). The primers used for cloning are listed in Supplementary Table S4.

### 4.5. Expression Level Analysis of Transgenic Plants

According to the above method, qRT-PCR is used to detect the expression level of the target gene in transgenic plants and WT plants. Total RNA is extracted from 1-month-old leaves and reverse transcribed into cDNA. Gene-specific primers are designed according to the gene sequence information of *BnCNGC57*, and PCR amplification is used to analyze the expression of the *BnCNGC57* gene. The *B. napus* F-box gene is used as an internal reference gene. All data are obtained from three biological replicates. The primers used for cloning are listed in Supplementary Table S4.

### 4.6. Phenotypic Analysis of Transgenic Plants

To evaluate the effect of *BnCNGC57* on the agronomic traits, both WT and overexpression plants were planted in a greenhouse. At least three plants were sampled for the following agronomic traits: seedling growth, root length, flowering data, flower size, 100-seed weight, and samples of transgenic plants and wild-type plants are taken. The second true leaves of one-month-old seedlings and 20 DAP seeds are quickly fixed with liquid nitrogen and stored at −80 °C for transcriptome sequencing. There are a total of 12 samples, with three replicates for each treatment.

### 4.7. RNA Extraction, Transcriptome Sequencing, and Differential Expression Analysis

Total RNA was extracted from the leaves and 20 DAP seeds of transgenic and wild-type (Westar) plants using an RNA extraction kit (Vazyme, Nanjing, China), and three biological repeats were performed for each sample. The concentration and quality of the extracted RNA were determined using the Agilent 2100 Bioanalyzer (Agilent, Santa Clara, CA, USA) and NanoDrop 2000 spectrophotometer (Thermo, Waltham, MA, USA), respectively, using only high quality RNA samples (OD_260_/OD_280_ ≈ 2.0~2.2, OD_260_/OD_230_ ≥ 2.0, RIN ≥ 8.0, 28S:18S ≥ 1.0) to construct sequencing libraries. After cDNA library construction, PE 100/150 bases reads are generated on the G400/T7/T10 platform (BGI-Shenzhen, China). The sequencing data were filtered with SOAPnuke (v1.5.6) [80] (https://github.com/BGI-flexlab/SOAPnuke, accessed on 21 August 2024). The clean reads were mapped to the reference genome of *B. napus* in NCBI (GCF_000686985. 2 _bra_napus_v2. 0) using HISTA2 (v2.1.0) [81] (https://daehwankimlab.github.io/hisat2/, accessed on 23 August 2024). Then, Ericscript (v0.5.5) [82] (https://sourceforge.net/projects/ericscript/, accessed on 24 August 2024) and rMATS (V3.2.5) [83] (http://rnaseq-mats.sourceforge.net, accessed on 24 August 2024) were used to detect fusion genes and differential splicing genes (DSGs), respectively. The Bowtie2 (v2.3.4.3) [84] (http://bowtie-bio.sourceforge.net/bowtie2/index.shtml, accessed on 25 August 2024) was used for the clean reads alignment to reference genome sequences, then the RSEM (v1.3.1) [85] (http://deweylab.biostat.wisc.edu/rsem/rsem-calculate-expression.html, accessed on 26 August 2024) was used to calculate the gene expression levels of all the samples. The heatmap was drawn by pheatmap (v1.0.8) (https://rdrr.io/github/raivokolde/pheatmap/, accessed on 26 August 2024) according to the gene expression difference in different samples. Essentially, differential expression analysis was performed using the DESeq2 (v1.4.5) [86] with Q value ≤ 0.05 (or FDR ≤ 0.05). Using clusterProfiler R packages through the GO database website (https://www.geneontology.org/, accessed on 27 August 2024) and KEGG automatic annotation server (KAAS, Separate the DEGs http://www.genome.jp/kegg/kaas/, accessed on 29 August 2024) Gene Ontology (GO) and Kyoto Encyclopedia of Genes and Genomes (KEGG) enrichment analysis were conducted [87,88].

### 4.8. Statistical Analysis

All data obtained were processed in SPSS, and statistical analysis was conducted by a one-way ANOVA or unpaired Student’s t-test. Significant differences were considered if *p*-values < 0.05.

## 5. Conclusions

In this study, 61 CNGC genes were identified in *B. napus* and categorized into four distinct groups. Comparative analysis of the collinearity among CNGC proteins in *B. napus*, *Arabidopsis*, and *B. rapa* offers a more nuanced understanding of the evolutionary history of *BnCNGC* genes. Furthermore, the research predicts the subcellular localization, 3D structure, and transmembrane domains of the CNGC family members in *B. napus*, while also examining the cis-acting regulatory elements within the promoter regions. In this investigation, an overexpression line of the *BnCNGC57* gene, which shares high homology with *AtCNGC2*, was successfully developed. Experimental outcomes indicate that the overexpression of *BnCNGC57* positively influences seed development, as evidenced by a significant increase in seed size. The potential mechanism behind the increased seed grain size due to *BnCNGC57* overexpression was explored at the transcriptomic level, revealing that it may regulate cell proliferation via the MAPK signaling pathway-plant, thereby promoting the enlargement of seed grain size.

## Figures and Tables

**Table 1:** *BnCNGC* evolutionary group member table.

Group Ⅰ		Group Ⅱ		Group Ⅲ		Group Ⅳ-a		Group Ⅳ-b	
Gene ID	Gene Name	Gene ID	Gene Name	Gene ID	Gene Name	Gene ID	Gene Name	Gene ID	Gene Name
BnaC03g31050D	BnCNGC1	BnaC05g12210D	BnCNGC16	BnaC04g16000D	BnCNGC26	BnaCnng45430D	BnCNGC38	BnaA10g18740D	BnCNGC56
BnaA04g15220D	BnCNGC2	BnaA06g10680D	BnCNGC17	BnaA07g13760D	BnCNGC27	BnaA03g34680D	BnCNGC39	BnaC09g42460D	BnCNGC57
BnaC04g38170D	BnCNGC3	BnaA02g07990D	BnCNGC18	BnaC09g42720D	BnCNGC28	BnaC03g40070D	BnCNGC40	BnaC09g30630D	BnCNGC58
BnaC06g16240D	BnCNGC4	BnaC02g11090D	BnCNGC19	BnaA10g19030D	BnCNGC29	BnaA03g34700D	BnCNGC41	BnaA10g07330D	BnCNGC59
BnaA07g17630D	BnCNGC5	BnaA03g50250D	BnCNGC20	BnaCnng12140D	BnCNGC30	BnaC01g34960D	BnCNGC42	BnaC02g44680D	BnCNGC60
BnaC06g16330D	BnCNGC6	BnaC07g42730D	BnCNGC21	BnaA06g16940D	BnCNGC31	BnaC05g35840D	BnCNGC43	BnaA02g09790D	BnCNGC61
BnaC04g01250D	BnCNGC7	BnaA04g14030D	BnCNGC22	BnaC01g08020D	BnCNGC32	BnaA05g22550D	BnCNGC44		
BnaA05g01380D	BnCNGC8	BnaC04g36890D	BnCNGC23	BnaA01g06670D	BnCNGC33	BnaA01g27850D	BnCNGC45		
BnaC03g31720D	BnCNGC9	BnaA09g41750D	BnCNGC24	BnaA09g41300D	BnCNGC34	BnaCnng48280D	BnCNGC46		
BnaA03g26780D	BnCNGC10	BnaCnng47090D	BnCNGC25	BnaC08g33890D	BnCNGC35	BnaA01g22170D	BnCNGC47		
BnaAnng17920D	BnCNGC11			BnaA04g14530D	BnCNGC36	BnaC01g34940D	BnCNGC48		
BnaA02g10440D	BnCNGC12			BnaC04g36270D	BnCNGC37	BnaC01g42610D	BnCNGC49		
BnaC02g14560D	BnCNGC13					BnaA05g22560D	BnCNGC50		
BnaA10g06480D	BnCNGC14					BnaC05g35850D	BnCNGC51		
BnaC09g29350D	BnCNGC15					BnaA05g22570D	BnCNGC52		
						BnaC05g35860D	BnCNGC53		
						BnaCnng48270D	BnCNGC54		
						BnaA01g27860D	BnCNGC55		

## Data Availability

The original contributions presented in the study are included in the article/Supplementary Materials, further inquiries can be directed to the corresponding author/s.

## Supplementary Materials

The supporting information can be downloaded at: https://www.mdpi.com/article/10.3390/ijms252111359/s1.
