# Supplementary material for: Genome Analysis of BnCNGC Gene Family and Function Exploration of BnCNGC57 in Brassica napus L
Source: Int J Mol Sci. 2024 Oct 22;25(21):11359. doi: 10.3390/ijms252111359 (PMC11545589; doi:10.3390/ijms252111359)
Supplement: Supplementary file 1 [file ijms-25-11359-s001.zip › Supplementary figures.pdf]

Supplementary files

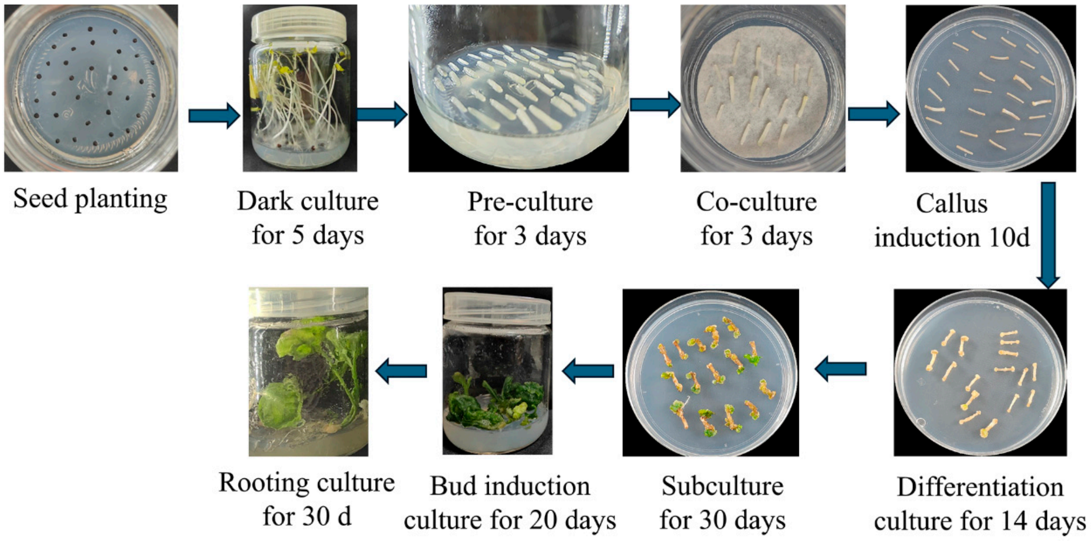

Supplementary Figure S1 The genetic transformation process of BnCNGC49 overexpression plants.

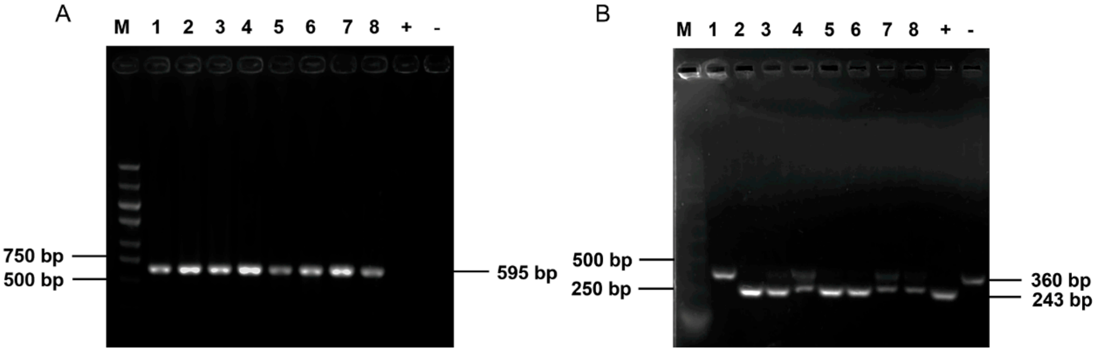

Supplementary Figure S2 Identification of transgenic lines of rapeseed BnCNGC49. (A) is the amplification fragment of Hyg gene, and (B) is the amplification fragment of BnaCNGC49 gene. Note: M is KM5000 DNA Marker. 1 is empty vector pTA7001-DEST. 2-3 are transgenic seedlings of pTA7001-BnaCNGC49. 4-8 are transgenic seedlings of pMDC83-BnaCNGC49. The positive control template "+" is pMDC83-BnaCNGC49 plasmid. The negative control "-" template is non-transgenic rapeseed. The specific amplification fragment size of BnaCNGC49 is 360 bp in gDNA and 243 bp in cDNA. The target fragment size amplified by hygromycin (Hyg) is 595 bp.

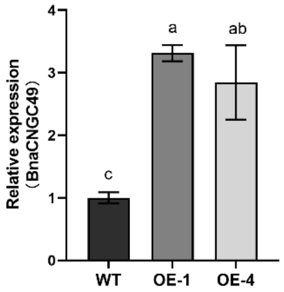

Supplementary Figure S3 Identification of gene expression levels in BnaCNGC49 overexpressing rapeseed lines. Note: “WT” is wild-type plant Westar, and “OE” is transgenic rapeseed with constitutive overexpression vector pMDC83-BnaCNGC49. Values and error bars represent mean ± SD (n = 3), and letters indicate significant differences by one-way ANOVA statistical analysis ($P < 0.05$).

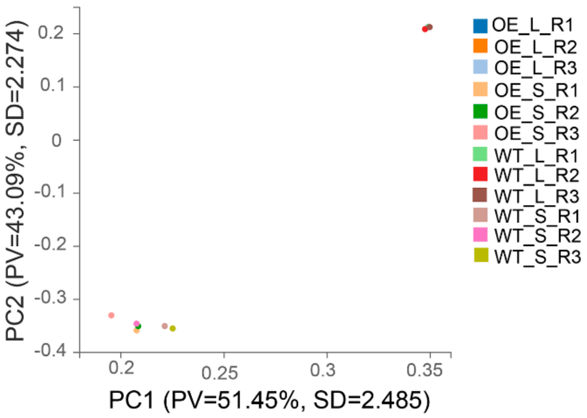

Supplementary Figure S4 Principal component analysis (PCA) plot showing the clustering of transgenic lines overexpressing BnCNGC57 (BnaC09g42460D).

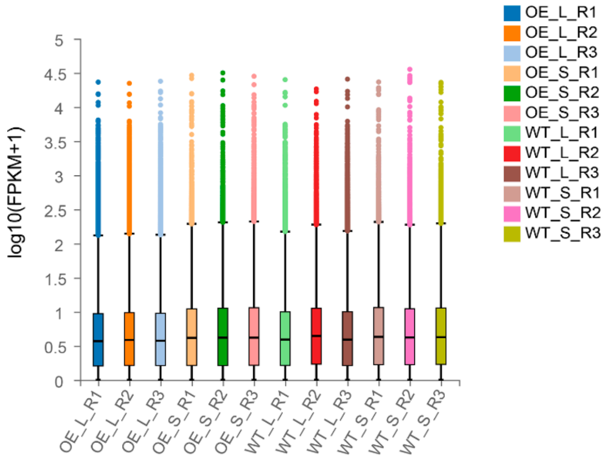

Supplementary Figure S5 Expression box line diagram showing the clustering of transgenic lines overexpressing BnCNGC57 (BnaC09g42460D).
